# Supplementary material for: Chromothripsis during telomere crisis is independent of NHEJ, and consistent with a replicative origin
Source: Genome Res. 2019 May;29(5):737–49. doi: 10.1101/gr.240705.118 (PMC6499312; doi:10.1101/gr.240705.118)
Supplement: Supplemental Material [file supp_gr.240705.118_Supplemental_file_1.zip › contigs/annotated_contigs/DB99/contig.2.DB99_length_390_mean_cov_16.5384615385.docx]

**DB99_length_390_mean_cov_16.5384615385**

```
CCAACTTGGGTCCATTCTCCTCATCACTTTCAGGTACACCAATCAGACGTAGATTTGGTCTGTTCACATAGTCCCGTATTTCTTGGAGT
 >chr3:94783244-94783397 + E=1e-77 p=0e+00
CTTTGTTCATTTCTTTTTATTCTTTTTTCTCTAAACTTCCCTTATCACTTCATTTCATTCATTT|AATAATACTTATAAT|ACTTAATA
                                                                                >chr3:9
ATACTTATAAAATCTCAACTCATACCAAGTTATCTTTTCTAACCAAAATTGAATTCTACTAGACATCAATAACATAAGAAGAACAGGAA
4787356-94787578 + E=3e-122
ATTTTCAACTAAGAAGAAATTAAGCAACACATCCTCACCCCTGAATAATCAATGAGTCAAAGAAAAAAATCGAAAGAAAAACTAACAGA

CACTTCTCAAAAGAAGACATTTATGAAGCCAAAAAA
```
